# Ultra-High Sensitivity Methane Gas Sensor Based on Cryptophane-A Thin Film Depositing in Double D-Shaped Photonic Crystal Fiber Using the Vernier Effect

**DOI:** 10.3390/s24248132

**Published:** 2024-12-19

**Authors:** Di Zhou, Sajid Ullah, Sa Zhang, Shuguang Li

**Affiliations:** State Key Laboratory of Metastable Materials Science & Technology and Key Laboratory for Microstructural Material Physics of Hebei Province, School of Science, Yanshan University, Qinhuangdao 066004, China; zhoudi@stumail.ysu.edu.cn (D.Z.); sajidktkphy@gmail.com (S.U.); 18332715635@163.com (S.Z.)

**Keywords:** D-shaped photonic crystal fiber, gas sensor, Sagnac interferometer, Vernier effect

## Abstract

Methane gas leakage can lead to pollution problems, such as rising ambient temperature. In this paper, the Vernier effect of a double D-shaped photonic crystal fiber (PCF) in a Sagnac interferometer (SI) is proposed for the accurate detection of mixed methane gas content in the gas. The optical fiber structure of the effective sensing in the sensing SI loop and the effective sensing in the reference SI loop are the same. Both of them adopt the polarization-maintaining photonic crystal fiber (PM-PCF) designed in this paper. The optical fiber structure of the effective sensing in the sensing SI loop deposited with the methane gas-sensitive film is polished to obtain a double-D structure. This operation makes it easier for methane gas to contact the sensitive film and realize the sensor’s repeated use. The sensing capability of the methane gas sensor was evaluated utilizing the finite element method (FEM). The numerical simulation results show that when the concentration of methane gas in the environment is 0~3.5%, the average sensitivity of two parallel Sagnac loops is 409.43 nm/%. Using Vernier effect cascade SI loops, the sensitivity of the sensor for detecting methane gas increased by four times. Without considering air and humidity, we provide a practical scheme for the development and design of high-sensitivity methane gas sensors.

## 1. Introduction

Optical fiber sensors are widely used in our lives [1,2]. They convert the state of the measured object into an observable optical signal and transmit information by analyzing changes in the output spectrum. With the innovation and development of science and technology, optical fibers are made into various devices, among which optical fibers are used more and more widely as sensors. In addition to measuring the most basic physical environment, such as magnetic fields, twists, and vibrations, they can also be applied to detect human health and in other related fields. Li et al. proposed a Sagnac interferometric high-sensitivity helical microstructured fiber (HMSF) directional twist sensor for twist measurement [3]. Wang et al. proposed an SI biosensor with microfiber. The adsorption of bovine serum albumin (BSA) molecules by graphene oxide was used to achieve ideal detection results [4].

Methane gas is a natural gas in our atmosphere. Methane is highly flammable and has security risks. Lelieveld J et al. showed that methane leakage into the atmosphere is below about 3%, and the use of natural gas as fuel should contribute least to climate forcing [5]. Therefore, the accurate measurement of methane gas is essential. Buse Comert et al. proposed a methane gas sensor prepared using TiO_2_ thin films with a particle size of 10 nm with high sensitivity and fast response/recovery time [6]. Rezvan Ghanbari et al. proposed that graphene is indeed decorated with silver nanoparticles (AgNPs) with an average size of 29.3 nm, distributed evenly. Sensors based on this material detect its response to the presence of methane gas [7]. Many researchers have conducted a series of studies on the application of optical fiber to this measurement. Liu et al. utilized the polarization filtering capabilities of photonic crystal fiber surface plasmon resonance (PCF-SPR). It can be used for detecting the content of methane gas in the external environment and detecting the content of hydrogen gas at the same time [8]. This approach allows for selective detection of specific gases, ensuring accurate measurements of both methane and hydrogen in mixed gases without interference. Chen et al. used carbon nanotubes doped with lithium ions to prepare electrode sensors, which achieved methane gas sensing by having a strong adsorption ability for methane [9]. The concentration of methane gas was detected. At 500 ppm, it reached a maximum of about 14.5%. Liu et al. conducted experiments on methane gas sensing using polarization-maintaining photonic crystal fiber (PM-PCF) Sagnac ring filters while eliminating the influence of cross gases on sensitivity [10]. Y.L. Hoo et al. proposed a hollow-core photonic bandgap fiber (HC-PBF) methane gas sensor [11]. The sensor type has a periodic microchannel structure, which can achieve high-sensitivity distributed sensing. Takaya Iseki et al. proposed a portable methane remote sensor made of 1.65 μm InGaAs laser and photodiode, which is compact in size and easy to use [12]. Crawford Massie et al. designed a sensor that was based on working around methane overtone absorption lines at 1660 nm using near-infrared LEDs [13]. It is a low-cost portable optical sensor for methane detection with a sensitivity of ∼1% of the Lower Explosive Level (LEL) for methane (500 ppm). Yang et al. simulated and experimented with a modal interference-based photonic crystal fiber (PCF) methane sensor with a good sensitivity of 0.514 nm/% [14]. Liu et al. simulated a highly sensitive transverse stress compensation methane sensor based on photonic crystal fiber long-period grating (PCF-LPG) with a methane gas sensitivity of 6.39 nm/% [15]. Yang et al. proposed a high-sensitivity long-period fiber grating (LPFG) methane sensor, and the experimental results show that the sensitivity is 2.5 nm/%, and the lowest detection limit is 0.2% [16]. Liu et al. designed a tellurate PCF sensor for simultaneous measurement of methane and hydrogen with a maximum sensitivity of 2.052 nm/% for methane gas and 0.236 nm/% for hydrogen gas [17]. As mentioned above, achieving ultra-high sensitivity remains a challenge for researchers to crack.

In this paper, an easy-to-implement guideline for improving the sensitivity of methane gas detection is proposed. It is composed of two parallel fiber SI rings in parallel and is obtained by using the Vernier effect. The polarization-maintaining photonic crystal fiber (PM-PCF) designed in this paper is used in the effective sensing part of the reference loop and the effective sensing part of the sensing loop. Making the part of the effective sensing in the sensing SI loop requires three steps. In the first step, the small air hole of the PCF is plugged. In the second step, the largest air hole of the PCF is deposited with methane gas-sensitive film by immersion technology. In the third step, the PCF is polished to the desired depth. The methane gas concentration is 0–3.5%, and the average sensitivity of two parallel Sagnac rings is 409.43 nm/%. Thus, it provides a practical design and guidance scheme for achieving high-sensitivity methane gas detection.

## 2. PCF Design and Numerical Analysis Methods

**Figure 1:**
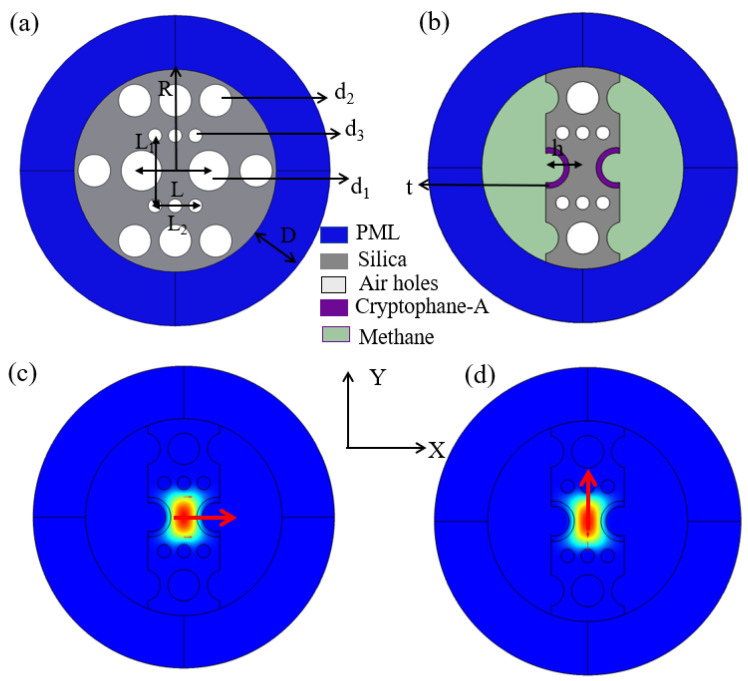
(**a**) The end-face view after PCF truncation; (**b**) PCF cross-section after polishing; (**c**) polarization mode X-direction electric field diagram; and (**d**) polarization mode Y-direction electric field diagram.

The simulation design sensor is optimized in five steps. First, the optimization of fiber optic structure in simulation design starts with the arrangement and radius of the holes. Second, the thickness of the methane-sensitive film deposited in two large air holes of PCF is optimized. Third, the largest pores of the fiber are polished to the best depth. The fourth step is to optimize the D-shaped PCF length of the SI sensing part. The fifth step is to optimize the PCF length of the SI reference part. Because PCF has processing errors in the actual preparation process, here, the structural parameters of PCF were not optimized, such as air hole arrangement, pore size, etc. The sensing SI loop uses a two-step filling technique to block small air holes and an immersion technique to deposit methane gas-sensitive film on large air holes in PM-PCF. After the above treatment of the fiber, the largest air hole is polished to find the best depth. This allows for better contact between the gas being measured and the PCF that the sensor effectively senses. Without changing the structure of the sensor, repeated measurement and multiple utilization can be achieved. The sensing part of the sensor after polishing saves labor and material costs and provides the possibility for actual mass production and a large number of applications. As depicted in Figure 1a, the end-face view after PCF truncation is depicted. High birefringence can be obtained by the asymmetrical arrangement of inner air holes in PCF. The two largest air holes, with d_1_ = 6 × 10^3^ nm, are in the x-axis direction. A layer of t-thick methane gas-sensing material is coated in the largest air holes. The center distance of the two largest air holes is L = 1 × 10^4^ nm. The outermost ring has eight air holes of d_2_ = 4.8 × 10^3^ nm. There are six air holes forming a regular hexagon, with two at the midpoint of the sides. There are six small air holes of d_3_ = 2 × 10^3^ nm in the inner layer, and the overall layout is rectangular. The long side of the rectangle is L_1_ = 6√3 × 10^3^ nm. The short side of the rectangle is L_2_ = 6 × 10^3^ nm. h is the distance from the remaining part of the fiber to the center after polishing, as shown in Figure 1b. The radius of the designed PCF is R = 1.5 × 10^4^ nm. We used the commercial software COMSOL (5.5) based on the finite element method (FEM) for numerical analysis simulation. A perfectly matched layer (PML) with a thickness of D = 8 × 10^3^ nm was applied in the external area of the PCF. PML acts as an almost ideal absorber, absorbing light signals that leave the core and radiate into the cladding to make the simulation more accurate and get the desired results. The electric field distributions in the x-direction and y-direction obtained by simulation calculation are shown in Figure 1c,d.

The optical fiber in the model is made of pure silicon dioxide, and its refractive index (RI) can be expressed using the Sellmeier formula:

(1) $$n^{2}(\lambda )=1+\sum_{i=1}^{3}\frac{A_{i}\lambda^{2}}{\lambda^{2}-{B_{i}}^{2}}$$

where λ is the wavelength of the incident light, *A*_1_ = 696.1663 nm, *A*_2_ = 407.9426 nm, *A*_3_ = 897.4794 nm, *B*_1_ = 68.4043 nm, *B*_2_ = 116.2414 nm, and *B*_3_ = 9896.161 nm [18]. Methane gas reacts with methane-sensitive membranes reversibly. When the concentration of methane gas in the external environment ranges from 0 to 3.5%, the values of the effective RI of methane-sensitive film change linearly. The values of the effective RI of methane-sensitive film increase by 0.0038 with each 1% decrease in methane concentration [14].

(2) $$n_{\mathrm{gas}}=1.4478\;-\;0.0038c$$

The optical fiber parameters *h* = 5 × 10^3^ nm and *t* = 500 nm will be studied and discussed below. The model is divided into 4083 domain elements and 441 boundary elements.

## 3. Discussion on Sensing Principles and Results

### 3.1. Single Sagnac Interferometer

**Figure 2:**
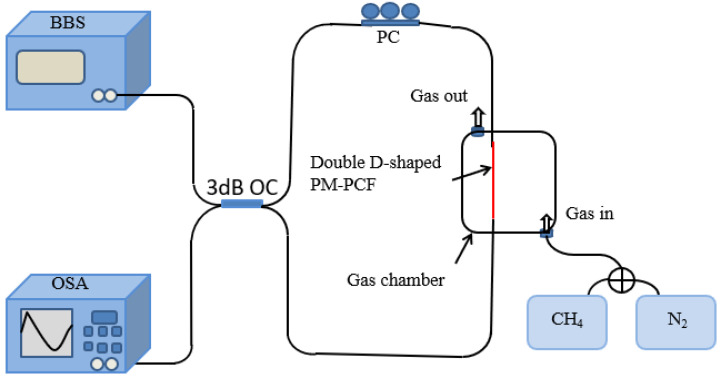
PCF methane gas-sensing principle drawing based on a single SI loop.

SI is a wave-dependent intensity modulation due to the phase difference between two orthogonal polarization modes of the x-axes and y-axes. The schematic drawing of the PCF methane gas sensing system with a single SI loop is displayed in Figure 2.

After the light passes through the 3 dB optical coupler (OC), the incident light generated by the broadband light source (BBS) is divided into two beams of equal energy. The two beams of light move towards each other in the SI loop, taking the same path but passing through the opposite polarization controller (PC) and the effective sensing region. The value of the effective RI of the methane gas-sensitive membrane changes with the concentration of the methane gas content in the external environment. The RI difference between the x-pol and y-pol of the PCF depositing the methane gas-sensitive film through the maximum air hole is constant. When they recombine into the 3 dB OC, they will be merged into one light beam. Eventually, it converges into the Optical Spectrum Analyzer (OSA) to obtain interference spectra. Methane gas is less dense and automatically moves upward. In order to make full contact between the effective sensing area and the methane gas in the gas chamber to be tested, the gas is injected from the bottom end of the gas chamber and discharged from the top of the gas chamber. The original gas is emptied into the gas chamber before passing the gas. By adjusting the ratio of methane gas to nitrogen gas, the concentration of methane gas in the gas chamber to be tested is determined. When two transmitted waves in opposite directions converge, the phase difference between them determines the interference intensity of a single SI loop. For the convenience of calculation, the normalized input light intensity is set to 1.

(3) $$I=\frac{1}{2}\left(1\;-\;\cos(\frac{2\pi \mathrm{BL}}{\lambda })\right),$$

Equation (3) means the output light intensity of the interference spectrum of SI. B = |n_x_ − n_y_| is the positive number, which means the absolute value of the birefringence between the X-direction and Y-direction polarizing base films of the PCF. It is a positive number. L is the length of the PCF, and λ represents the wavelength of the light corresponding to the entry into the PCF. When output light interference intensity is 0, the superposition of two beams of light appears to have coherent cancellation, resulting in an interference valley. The position of the interference valley in the transmission spectrum is λ = LB/m. m is an integer. When researching SI, it is indispensable to study the free spectral range of the interference spectrum (FSR). FSR = λ^2^/LB. Changes in the concentration of the methane gas in the external environment will be detected as it increases or decreases, and the birefringence value of the polarization fundamental mode will change due to the photoelastic and thermal effects of the fiber optic material, which can affect the wavelength of the interference spectrum to the left or to the right drift. By tracking the appropriate valley position movement, the effect of parameter sensing can be achieved, and the sensitivity of sensors can be expressed by Equation (4).

(4) $$S=\frac{d\lambda }{\mathrm{dC}}=\frac{\frac{\mathrm{dL}}{\mathrm{LdC}}B\left(\lambda ,C\right)\lambda \left(C\right)+\lambda \left(C\right)\frac{\partial B\left(\lambda ,C\right)}{\partial C}}{B\left(\lambda ,C\right)-\lambda \frac{\partial B\left(\lambda ,C\right)}{\partial C}},$$

**Figure 3:**
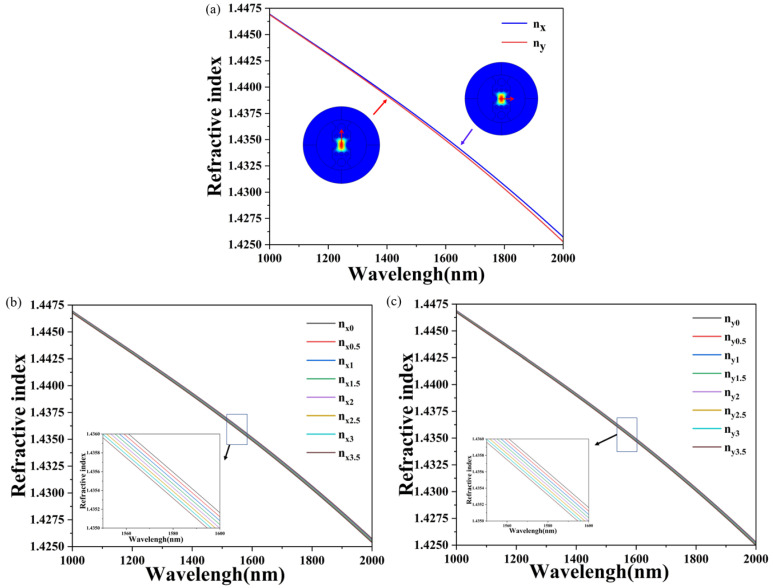

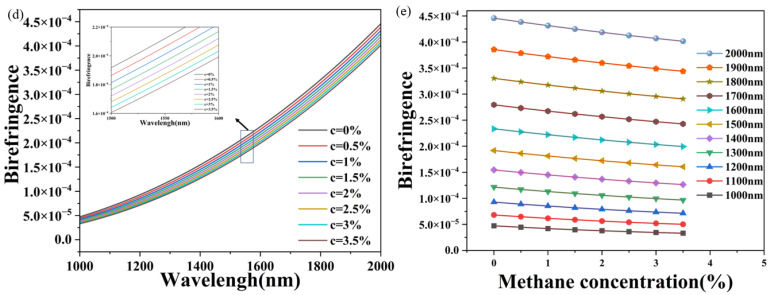
Trend image of refractive index (**a**) in the X direction and Y direction, (**b**) in the X direction, (**c**) in the Y direction of PCF, (**d**) trend image of phase birefringence B and wavelength at 0–3.5% methane concentration in the external environment, and (**e**) trend image of phase birefringence B versus methane concentration at wavelengths of 1000 to 2000 nm.

The simulation results are shown in Figure 3. B is related to wavelength and methane gas concentration. In Figure 3a, the blue line indicates the effective RI of the polarization mode in the X-direction, and the red line indicates the effective RI of the polarization mode in the Y-direction when the methane gas concentration is 0%. Figure 3b,c show the effective RI of two polarization modes in the X-direction and Y-direction of the PCF at 0–3.5% methane concentration in the external environment. B = |n_x_ − n_y_|. Figure 3d shows that the absolute value of the birefringence difference between two orthogonal polarization modes of the PCF increases with increasing wavelength. The larger the birefringence value of the PCF that is transmitted independently by two orthogonal polarization modes, the better the polarization preservation performance of the PCF. Under the condition that the wavelength of the incident light is the same, the absolute value of the birefringence difference in X-direction and Y-direction polarizing films decreases as methane gas concentration increases. As can be seen from Equation (2), the coefficient before gas concentration is −1, so RI will decrease with the rise in methane gas concentration in the environment. The birefringence values corresponding to seven different methane gas concentrations on the same value Y-axis in Figure 3d were fitted in Figure 3e. Figure 3e shows that the results of the fitting equations are B_1_ = 4.640 × 10^−5^ − 4.038 × 10^−6^x, B_2_ = 6.710 × 10^−5^ − 5.097 × 10^−6^x, B_3_ = 9.177 × 10^−5^ − 6.117 × 10^−6^x, B_4_ = 1.205 × 10^−4^ − 7.089 × 10^−6^x, B_5_ = 1.534 × 10^−4^ − 8.012 × 10^−6^x, B_6_ = 1.906 × 10^−4^ − 8.886 × 10^−6^x, B_7_ = 2.322 × 10^−4^ − 9.713 × 10^−6^x, B_8_ = 2.784 × 10^−4^ − 1.050 × 10^−5^x, B_9_ = 3.291 × 10^−4^ − 1.124 × 10^−5^x, B_10_ = 3.844 × 10^−4^ − 1.194 × 10^−5^x, and B_11_ = 4.456 × 10^−4^ − 1.261 × 10^−5^x. The linear fitting linearities were 0.987, 0.990, 0.992, 0.994, 0.995, 0.996, 0.996, 0.997, 0.997, 0.998, and 0.997, respectively. The absolute value of the birefringence difference between the X-direction and Y-direction polarizing film changes with the concentration of methane gas in the environment under different wavelength conditions, showing a decreasing trend.

**Figure 4:**
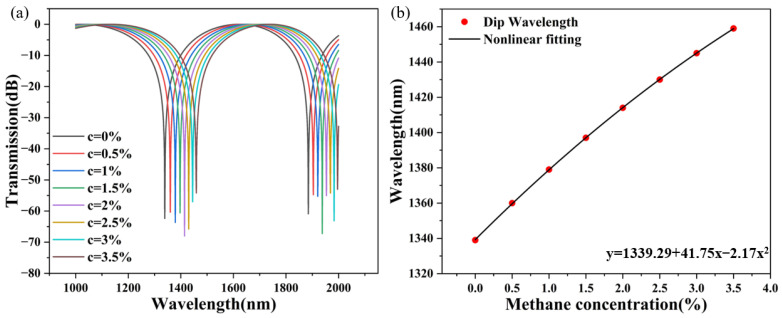
(**a**) The transmission spectrum of 500 nm methane gas sensing film with the concentration of methane gas 0–3.5% in the external gas environment and (**b**) the dip wavelength is fitted by a polynomial with methane concentration of 0–3.5%.

**Figure 5:**
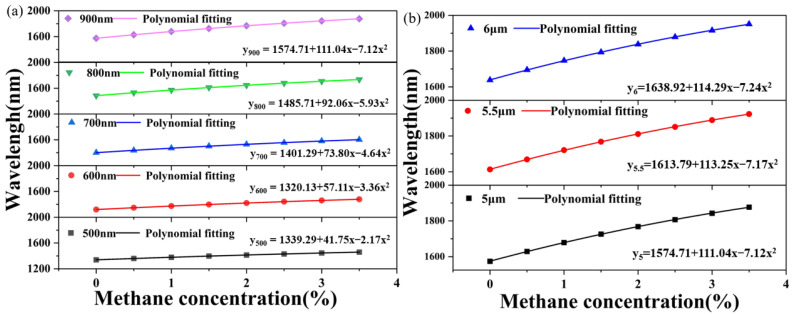
(**a**) The trend diagram of the interference inclination of the transmission spectrum and the methane concentration with the thickness of the methane-sensitive film increases from 500 nm to 900 nm and (**b**) the trend diagram of the interference inclination of the transmission spectrum and the methane concentration with the depth of polishing increases from 5 × 10^3^ nm to 6 × 10^3^ nm.

The simulation results are shown in Figure 4. In Figure 4a, interference valleys can be observed in the wavelength range of incident light of 1000~2000 nm. With the increase in methane concentration at 0.5/% intervals, the wavelength of the interference valley in the transmission spectrum will be red-shifted. With the increase in the incident wavelength, the distance between adjacent interference valleys in the transmission spectrum becomes smaller, and the distance between adjacent interference valleys becomes closer. That is to say, the gas sensitivity of the PCF sensor decreases with increasing wavelength. The position of the interference valley in the transmission spectrum is λ = LB/m. S = dλ/dC = L∂λ∂B/m∂B∂C. As with the trend change shown in Figure 3d, its slope ∂B/∂λ is greater than 0. The slope increases with the increase in wavelength λ. The reciprocal ∂λ/∂B is also greater than 0 and decreases as λ increases. As with the trend change shown in Figure 3e, its slope ∂B/ ∂C is less than 0, and with the increase in the concentration c of methane gas in the external gas environment, the curve tends to be gentle, and the slope decreases. Because ∂λ/∂B is greater than 0 and ∂B/ ∂C is less than 0, multiplying the two together is a negative number, and the sensitivity of the largest air hole deposition gas-sensitive film of the PCF methane gas sensor is negative. We fit the seven interference valley wavelengths using the linear fitting equation y = 1339.29 + 41.75x − 2.17x^2^, with a linearity of 0.99998 and an average sensitivity of 34.28 nm/% in Figure 4b. Since it is difficult to change the original radius of air holes in stretched PCF, we have chosen to investigate the thickness of the methane-sensitive film and the depth of the polishing. Because of the methane gas-sensitive film deposited on the largest air hole of the PCF, the thickness of the film cannot be increased indefinitely, which is affected by the hole structure. The changing trend of the transmission and wavelength of methane gas-sensing films deposited in the largest hole of PCF at 600 nm, 700 nm, 800 nm, and 900 nm are calculated. The wavelength sensitivity is affected by the thickness of the largest hole in the PCF. As the thickness of the film increased, the change in sensitivity was positively correlated and also increased, as shown in Figure 5a; the simulation results are represented by polynomial fitting. The fitting parameters under different film thicknesses are shown in Table 1. From Figure 5a and Table 1, we determined that the film thickness was 900 nm.

The depth of the PCF polishing is discussed. If the polishing depth of the PCF is not sufficient, the gas cannot directly enter the effective sensing part of the PCF, and it cannot react with the gas-sensitive film. If the PCF is polished too deeply, the PCF becomes too thin and easily becomes fragmented, resulting in overall damage to the sensor. As the polishing depth of PCF increases, the change in sensitivity was positively correlated and also increased, as shown in Figure 5b; the simulation results are represented by polynomial fitting. The fitting parameters under different depths of polishing are shown in Table 2.

It can be seen that depth has little effect on wavelength sensitivity, which is on the order of one digit. Because the remaining PCF after polishing will become thinner, more remaining PCF with h = 6 × 10^3^ nm is selected, the remaining methane gas-sensitive film is the most, and the wavelength sensitivity is the highest, as discussed above. The double D-shaped PCF cascade with h = 6 × 10^3^ nm and t = 900 nm was selected to form SIs using the Vernier effect.

### 3.2. Two Parallel Sagnac Interferometers

**Figure 6:**
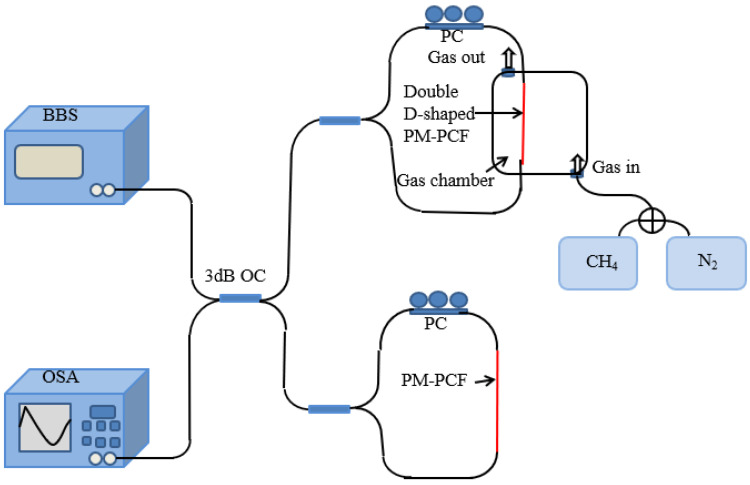
Deposition of methane gas-sensitive film on sensing arm based on Vernier effect reference arm without any modification of methane gas-sensing principle diagram.

The principle of the Vernier effect in optics is similar to the Vernier caliper technique used in physical measurement. The Vernier effect can be formed by the spectrum generated by the interferometers composed of two SI rings. The schematic diagram of the PCF methane gas-sensing system with a cascaded SI loop is displayed in Figure 6. The BBS produces the incident light, which is divided into two same-energy beams by the first 3 dB OC. One acts as a sensing SI loop to change the methane gas concentration parameter, while the other acts as a reference SI loop for an unmodified and polished PCF that never changes the parameter. The Vernier effect of an optical interference system depends on the combination of two interferometers, and the overlap of two signals after concatenation produces envelope modulation. Two interference systems with different FSRS are cascaded together so that a Vernier effect can be introduced into the transmission spectrum, thereby stimulating an envelope spectrum with a larger FSR, which can greatly improve the sensitivity of the sensor. The combined light intensity resulting from the output light from the superposition of the two SI rings in the reference and sensing arms is expressed in Equation (5).

(5) $$I_{p}=I_{r}+I_{s},$$

Among them, Ir and Is are the intensities of the optical signal output by the SI loop of the reference and the sensing, respectively. The free spectral range generated by parallel SIs after using the Vernier effect can be described by Equation (6) [19].

(6) $${\mathrm{FSR}}_{\mathrm{enve}}=\frac{{\mathrm{FSR}}_{r}{\times \mathrm{FSR}}_{s}}{\left|{\mathrm{FSR}}_{r}{-\mathrm{FSR}}_{s}\right|},$$

The magnification factor (M) is a very significant physical quantity to describe the effect of the Vernier effect in optics, as shown in Equation (7).

(7) $$M=\frac{{\mathrm{FSR}}_{\mathrm{enve}}}{{\mathrm{FSR}}_{s}}=\frac{{\mathrm{FSR}}_{r}}{\left|{\mathrm{FSR}}_{r}{-\mathrm{FSR}}_{s}\right|}$$

**Figure 7:**
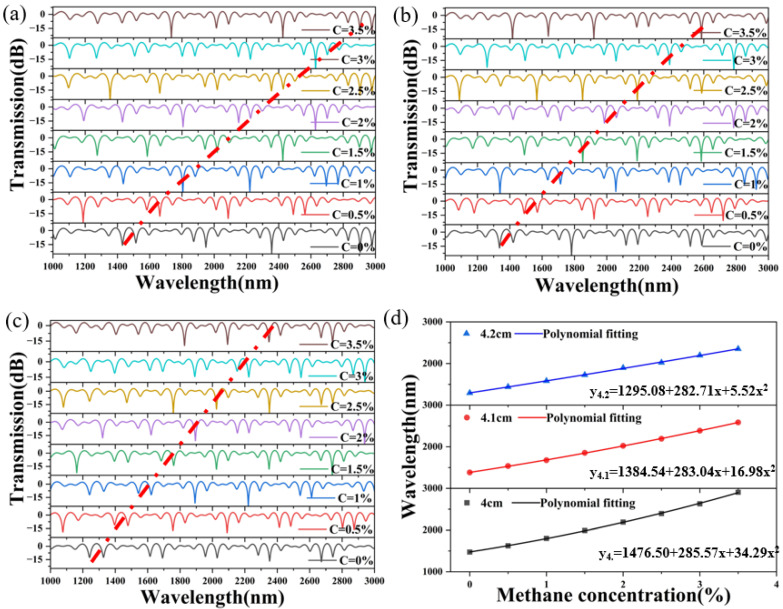
(**a**) The transmission spectrum of the reference SI loop is 4 cm in length, (**b**) the transmission spectrum of the reference SI loop is 4.1 cm in length, and (**c**) the transmission spectrum of the reference SI loop is 4.2 cm in length between 1000 and 3000 nm in wavelength. (The red line in (**a**–**c**) is the interference valley tracking curve) (**d**) The image is a polynomial fitting of the inclination wavelength of the transmission spectrum to the content of the external methane concentration.

To form a short, compact, and portable sensor, the lengths of the effective sensing part of the sensor loop and the reference loop are not much different, and a sensor loop of the same size is formed. Because the PCF of the sensing loop is deposited and polished, the PCF becomes very brittle, so we decided to give a fixed value of 10 for the effective sensing length of the sensing loop. The PCF of the reference loop is not processed at all, and it is easier to replace a PCF of different lengths. As shown in Figure 7, the effective length of the PCF in the sensing SI loop is 1 × 10^8^ nm, and the effective length of the PCF in the reference SI loop is 4 × 10^7^ nm, 4.1 × 10^7^ nm, and 4.2 × 10^7^ nm. The interference spectrum using the Vernier effect is applied in the range of a methane gas concentration of 0–3.5%. The fitting parameters under different effective lengths of PCF in the reference SI loop are shown in Table 3.

**Figure 8:**
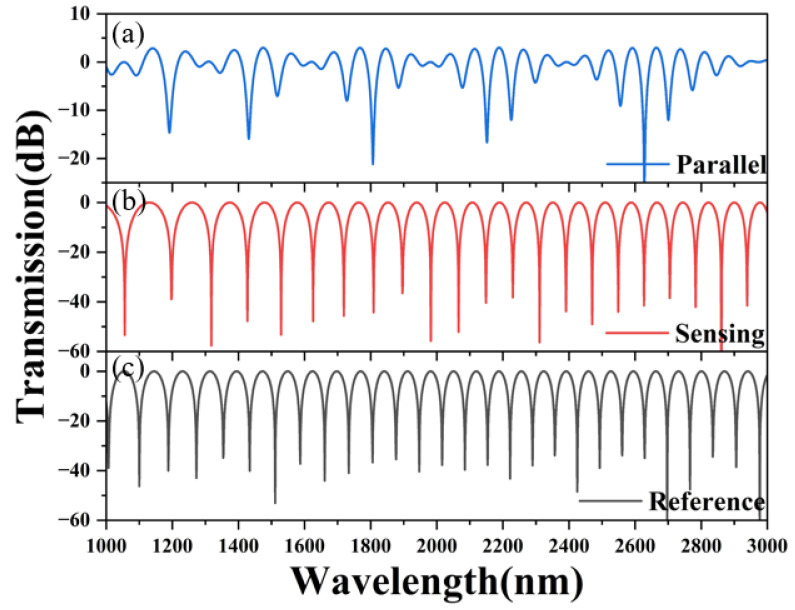
(**a**) The transmission spectrum of SI loops, (**b**) the transmission spectrum of the sensing SI loop is 1 × 10^8^ nm in length, and (**c**) the transmission spectrum of the reference SI loop is 4 × 10^7^ nm in length.

To excite the envelope spectrum, we need to induce subtle differences in the FSR of the two arms by varying the length of the PCF effective sensing in the sensor-sensing SI loop and reference SI loop. The simulation results show that the envelope of the transmission spectrum collectively red-shifts with increasing concentration of methane in the outside gas [20,21]. This produces a positive m factor. M = FSRr/|FSRr-FSRs| = 66/|66 − 80| = 4.71, as shown in Figure 7. The methane gas sensor with a selected methane gas-sensitive film thickness of 900 nm, sensing arm length of 1 × 10^8^ nm, and reference arm length of 4 × 10^7^ nm is shown in Figure 8.

**Figure 9:**
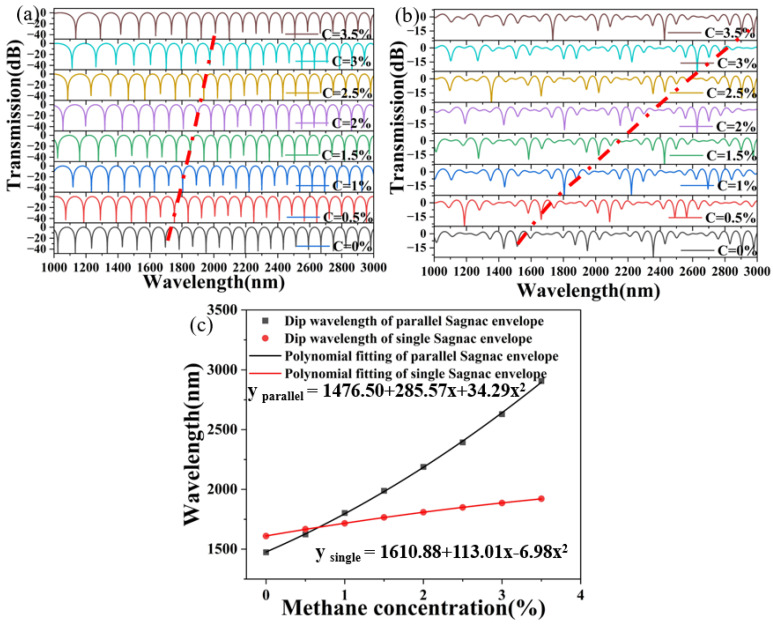
(**a**) The transmission spectrum of a single SI loop, and (**b**) the transmission spectrum of a parallel SI loop between 1000 and 3000 nm in wavelength. (The red line in (**a**,**b**) is the interference valley tracking curve). (**c**) The position of the two envelope wavelengths is fitted to the polynomial of the methane concentration.

Figure 9 shows the interference spectra of the PCF effective sensing in the sensing SI loop, the PCF effective sensing in the reference SI loop, and the introduction of the Vernier effect at a methane concentration of 2%. The FSR of the PCF effective sensing in the sensing SI loop is 81 nm, the FSR of the PCF effective sensing in the reference SI loop is 70 nm, and the FSR of the cascaded interference spectrum after introducing the Vernier effect is 468 nm. As shown in Figure 9, the largest air hole inner wall deposition of methane gas-sensitive film had a thickness of 900 nm when the PCF was used as the sensing arm, the wavelength sensitivity of a single Sagnac ring varies in the range of 0–3.5% with external methane concentration, and the fitting equation is y_single_ = 1610.88 + 113.01x − 6.98x^2^, with a linear degree of 0.99990 and an average sensitivity of 89.14 nm/%. The fitting equation for the cascaded wavelength sensitivity with the application of the Vernier effect is y_parallel_ = 1476.50 + 285.57x + 34.29x^2^, with a linear degree of 0.99971 and an average sensitivity of 409.43 nm/%. Compared with previous work, as shown in Table 4, we found that our fiber optic gas sensor has higher sensitivity and a more convenient method [22]. The effect of methane gas should be viewed in two parts: positive and negative. Methane is a carbon source that contributes to the growth and metabolism of plants and animals, synthesizing a variety of chemical materials. However, excess methane gas can lead to mine gas explosions and biological breathing difficulties that make it difficult for hemoglobin to bind to oxygen. Therefore, the accurate detection of methane gas plays an important role in low-carbon environmental protection.

## 4. Conclusions

This paper presents a practical design scheme for improving the sensitivity of a methane gas sensor. The sensor is based on two parallel-fiber SI loops utilizing the Vernier effect. Both the PCF effective sensing in the reference SI loop and the PCF effective sensing in the sensing SI loop are based on the PM-PCF designed in this paper. The methane gas-sensing performance of the sensor is verified by numerical analysis and finite element analysis. The sensor can realize high-sensitivity monitoring in the range of 0–3.5% outside methane concentration. The sensor of a single SI loop is analyzed. The sensitivity of gas detection increases with the increase in the thickness of the methane gas-sensitive film deposited on the largest air hole of the PCF. The film thickness cannot increase indefinitely. A gas-sensitive film that is too thick will affect the effective contact area of the gas and gas-sensitive film reaction. The depth of polishing has little effect on the sensitivity of gas detection. Based on the consideration of robustness, under the premise of satisfying the direct contact between the gas and the sensor, more optical fiber structure is retained. The sensitivity of a single SI loop at t = 900 nm and h = 6 × 10^3^ nm is 89.14 nm/%. Because the PCF after deposition and polishing treatment is fine and fragile, it is not easy to replace. Therefore, under the premise of the effective PCF length fixing method in the sensing loop, the PCF length without any modification treatment in the reference loop is fine-tuned and optimized. The PCF modified by type D is a sensing loop of effective sensing length, and the PCF without any treatment is a reference loop of effective reference length, using the Vernier effect. The sensitivity reaches 409.43 nm/%. This is a good and practical method. If the method is realized in practical applications, this methane gas sensor provides a feasible scheme for wide application in the process of natural gas exploitation and exploration. Our next step is to focus on the simultaneous measurement of multiple combustible gases based on high sensitivity.

## Figures and Tables

**Table 1:** Polynomial fitting of valley wavelength at different methane gas-sensitive film thicknesses.

y = a + b_1_ × x − b_2_ × x^2^					
t	Parameters				
	a	b_1_	b_2_	R^2^	Average Sensitivities (nm/%)
t = 500 nm	1339.29	41.75	2.17	0.99998	34.28
t = 600 nm	1320.13	57.11	3.36	0.99999	45.42
t = 700 nm	1401.29	73.80	4.64	0.99996	57.71
t = 800 nm	1485.71	92.06	5.93	0.99995	71.71
t = 900 nm	1574.71	111.04	7.12	0.99995	86.57

**Table 2:** Polynomial fitting of valley wavelength at different depths of polishing.

y = a + b_1_ × x − b_2_ × x^2^					
h	Parameters				
	a	b_1_	b_2_	R^2^	Average Sensitivities (nm/%)
h = 5 × 10^3^ nm	1574.71	111.04	7.12	0.99996	86.57
h = 5.5 × 10^3^ nm	1613.79	113.25	7.17	0.99995	88.57
h = 6 × 10^3^ nm	1638.92	114.29	7.24	0.99995	89.43

**Table 3:** Polynomial fitting of valley wavelength at different effective lengths of PCF in the reference SI loop.

y = a + b_1_ × x+ b_2_ × x^2^					
Effective Lengths of PCF in the Reference SI Loop	Parameters				
	a	b_1_	b_2_	R^2^	Average Sensitivities (nm/%)
4 × 10^7^ nm	1476.50	285.57	34.29	0.99971	409.43
4.1 × 10^7^ nm	1384.54	283.04	16.98	0.99983	343.71
4.2 × 10^7^ nm	1295.08	282.71	5.52	0.99956	302.29

**Table 4:** Performance comparison of various gas sensors.

Type of Sensor	Sensitivity	Method	Gas	Reference
PCF	0.514 nm/%	Experiment with methane and nitrogen mixed gas	methane	[14]
PCF-LPFG	2.5 nm/%	Experiment with methane and nitrogen mixed gas	methane	[16]
PC micro-cavity	1.67 nm/%	Simulation	methane	[23]
LPFG-SPR	0.344 nm/%	Experiment with methane and nitrogen mixed gas	methane	[24]
PCF-LPG	1.078 nm/%	Experiment with methane and nitrogen mixed gas	methane	[25]
PCF	36.64 nm/%	Simulation with methane and nitrogen mixed gas	methane	[26]
PCF-SPR	1.99 nm/%	Simulation with methane and hydrogen mixed gas	methane	[8]
PQF-SPR	8 nm/%	Simulation	methane	[27]
Tellurite-PCF	2.052 nm/%	Simulation with methane and hydrogen mixed gas	methane	[17]
PCF-LPG incorporating cryptophane-A	6:39 nm/%	Simulation with methane gas	methane	[15]
PCF	409.43 nm/%	Simulation with methane and nitrogen mixed gas	methane	This work

## Data Availability

The data that support the findings of this study are available upon reasonable request from the authors.
